# Higher-order correction of persistent batch effects in correlation networks

**DOI:** 10.1093/bioinformatics/btae531

**Published:** 2024-09-03

**Authors:** Soel Micheletti, Daniel Schlauch, John Quackenbush, Marouen Ben Guebila

**Affiliations:** Department of Biostatistics, Harvard T.H. Chan School of Public Health, Boston, MA 02115, United States; Department of Biostatistics, Harvard T.H. Chan School of Public Health, Boston, MA 02115, United States; Department of Data Science, Dana-Farber Cancer Institute, Boston, MA 02215, United States; Genospace, LLC, Boston, MA 02108, United States; Department of Biostatistics, Harvard T.H. Chan School of Public Health, Boston, MA 02115, United States; Department of Data Science, Dana-Farber Cancer Institute, Boston, MA 02215, United States; Channing Division of Network Medicine, Brigham and Women's Hospital, Boston, MA 02115, United States; Department of Biostatistics, Harvard T.H. Chan School of Public Health, Boston, MA 02115, United States

## Abstract

**Motivation:**

Systems biology analyses often use correlations in gene expression profiles to infer co-expression networks that are then used as input for gene regulatory network inference or to identify functional modules of co-expressed or putatively co-regulated genes. While systematic biases, including batch effects, are known to induce spurious associations and confound differential gene expression analyses (DE), the impact of batch effects on gene co-expression has not been fully explored. Methods have been developed to adjust expression values, ensuring conditional independence of mean and variance from batch or other covariates for each gene, resulting in improved fidelity of DE analysis. However, such adjustments do not address the potential for spurious differential co-expression (DC) between groups. Consequently, uncorrected, artifactual DC can skew the correlation structure, leading to the identification of false, non-biological associations, even when the input data are corrected using standard batch correction.

**Results:**

In this work, we demonstrate the persistence of confounders in covariance after standard batch correction using synthetic and real-world gene expression data examples. We then introduce Co-expression Batch Reduction Adjustment (COBRA), a method for computing a batch-corrected gene co-expression matrix based on estimating a conditional covariance matrix. COBRA estimates a reduced set of parameters expressing the co-expression matrix as a function of the sample covariates, allowing control for continuous and categorical covariates. COBRA is computationally efficient, leveraging the inherently modular structure of genomic data to estimate accurate gene regulatory associations and facilitate functional analysis for high-dimensional genomic data.

**Availability and implementation:**

COBRA is available under the GLP3 open source license in R and Python in netZoo (https://netzoo.github.io).

## 1 Introduction

Batch effects in gene expression analyses arise because samples are typically collected and processed in different groups or batches. If not properly corrected, batch effects can introduce artifacts that obscure the true biology driving the phenotypic differences that one hopes to study. Various methods have been developed to correct for batch effects in differential expression analysis, including ComBat (Johnson *et al.* 2007, Zhang *et al.* 2020), dChip (Li and Wong 2003), and more general procedures such as LIMMA (Ritchie *et al.* 2015) and SVA (Leek *et al.* 2012). Although these methods differ in implementation, each essentially performs a linear correction of batch effects, or “location-scale correction,” that adjusts the mean and variance of gene expression, improving the detection of biologically meaningful patterns of differential expression.

However, the factors driving phenotypic differences go beyond individual genes and instead involve sets (or networks) of genes that are coordinately activated or regulated to carry out specific functions—processes that can be missed when analyzing differential gene expression alone (Schlauch *et al.* 2017). Network science methods often consider the pairwise joint distribution of genes by creating and comparing co-expression networks to identify functional groups of genes that are coordinately expressed. These methods generally first perform standard batch correction, compute correlation-based co-expression matrices using the corrected expression data, and then compare the resulting networks between conditions. However, this does not account for the possibility of batch-induced correlations between genes, as standard batch correction methods act solely on the marginal distribution of each gene.

**Figure 1.**
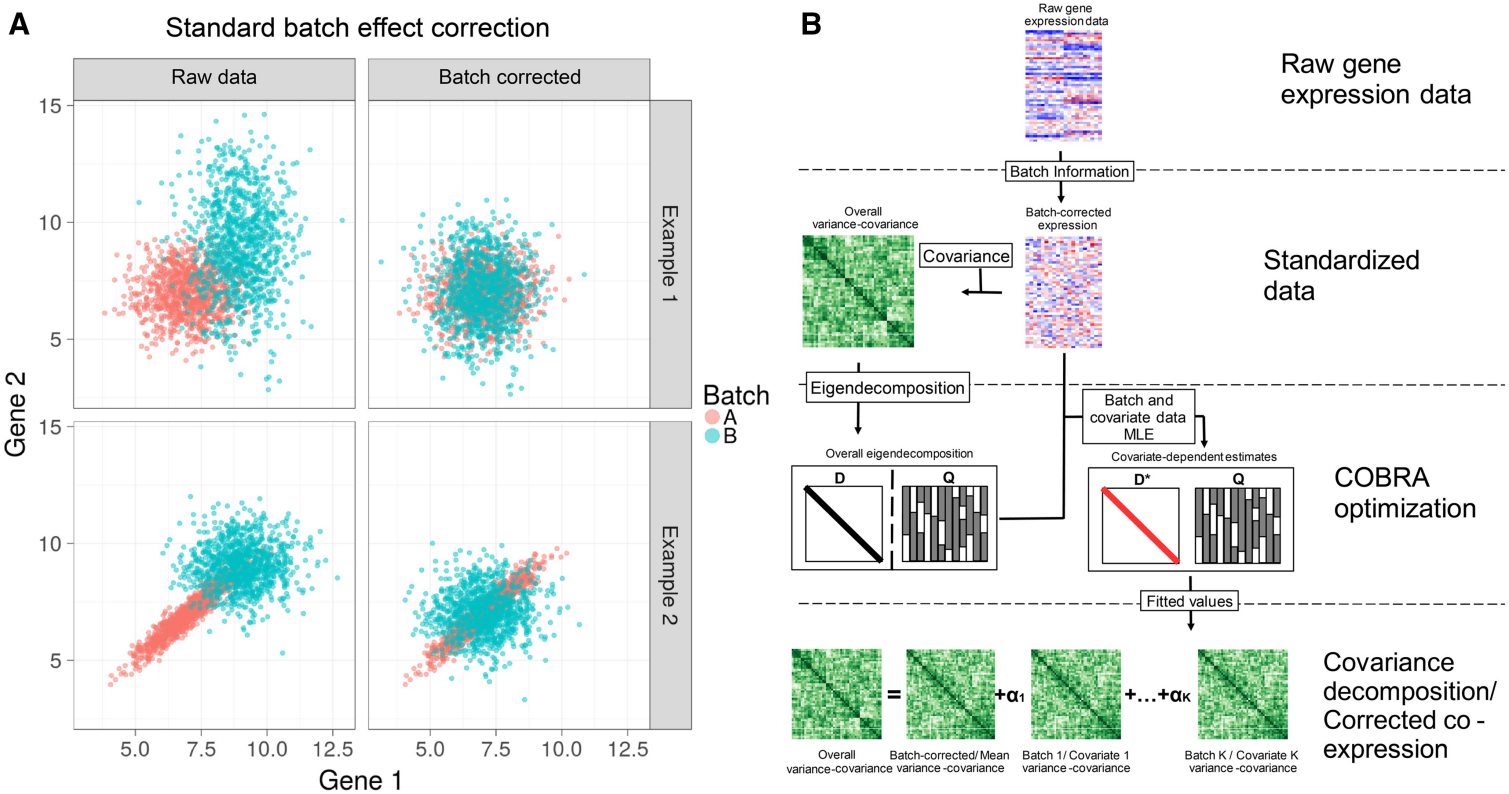
Identifying and correcting batch effects in correlation networks using COBRA. (A) Effect of standard batch correction on co-expression. Top: two conditionally independent genes simulated in two batches showing how batch effects can be corrected using ComBat’s location-scale method. Bottom: simulation of two artifactually correlated genes in Batch A but not Batch B; batch-dependent correlation persists after ComBat correction. (B) COBRA’s Workflow begins with (1) normalizing the input gene expression and (2) computing the co-expression matrix. (3) Performing an eigendecomposition of the co-expression matrix, and, using the eigenvectors as a basis, estimating “pseudo-eigenvalues” for each covariate, minimizing the reconstruction error. Finally, (4) the fitted pseudo-eigenvalues, in combination with the eigenvector matrix Q, are used to infer batch-corrected networks or to analyze covariate-specific co-expression.

As an example, consider a system of two simulated genes, each of which is profiled across multiple samples that are grouped into two batches (Fig. 1A). First, we consider a situation in which the expression of these genes is uncorrelated in both Batch A and Batch B, although with different batch-specific means and variances (upper left). As can be seen, batch correction using ComBat removes these differences and recovers their overall uncorrelated structure (upper right), so that the batches become, as expected, indistinguishable. Now, consider a situation where an artifactual correlation in the expression of Gene 1 and Gene 2 appears in Batch A but not Batch B (lower left); ComBat batch correction centers the data from both batches, but fails to address the correlation structure that appeared in Batch 1 (lower right). Such a failure to address this higher-order artifact can lead to spurious results in downstream analyses, particularly in applications that rely on correlation-based networks.

While some methods have addressed modeling the covariance matrix for small numbers of variables (Zou *et al.* 2017), extending these approaches to higher-dimensional biological data, such as genome-scale transcriptional data, is nontrivial. First, the sample covariance matrix can be singular and non-positive-definite. Second, estimating the co-expression matrix involves inferring a large number of parameters. Given *p* genes, there are $\left(\begin{array}{c} p\\ 2 \end{array}\right)$ pairwise correlations, each of which may be a function of the number of covariates in a particular experiment. In high-throughput gene expression studies, the number of genes *p* is typically much larger than the number of samples *n*, and one often finds that co-expression in the same system does not consistently replicate across studies, likely because of the large numbers of parameters that must be estimated (Schlauch *et al.* 2017). Imposing sparsity on the gene covariance matrix or precision matrix has been suggested as a possible way to address this issue (Friedman *et al.* 2008), but this is not well suited for gene expression data where there is no *a priori* biologically motivated justification for excluding some subset of genes.

Co-expression Batch Reduction Adjustment (COBRA) addresses the problem of batch effects in gene co-expression by leveraging the modular nature of gene expression data. COBRA effectively reduces the parameter space, estimating O(n) variables for each covariate, performing a dimensional reduction that allows estimation of the gene co-expression matrix as a function of sample covariates (which can be both continuous and categorical). COBRA can be applied for batch correction and covariate-specific co-expression analysis, both for Pearson correlation and other measures of association.

## 2 Materials and methods

### 2.1 Overview of the statistical approach

COBRA takes as input a gene expression matrix for *p* genes in *n* samples, and a design matrix *X* of dimension *n *×* q* containing a vector of *q* covariates for each sample. COBRA decomposes the co-expression matrix as a linear combination of components, one for each covariate in *X*, which can be used to correct for higher-moment batch effects in correlation matrices beyond biases in the mean and variance. By using the corrected co-expression matrix in network inference methods, we can effectively remove batch effects or identify covariate-specific co-expression patterns. In this section, after presenting our model and deriving an optimal estimator, we show how to correctly design *X* to tackle various problems.

### 2.2 Addressing persistent high-order batch effects using COBRA

Consider a set of *n* samples, for which we measure *q* covariates and the expression of *p* genes. Let $X_{i}:={\left(X_{i,1},\ldots ,X_{i,q}\right)}^{T}$ represent the covariates for sample *i*, and let $g_{i}:={\left(g_{i,1},\ldots ,g_{i,p}\right)}^{T}$ denote the gene expression values for sample *i* across the *p* genes. Moreover, let $G:=\left[g_{1}-\bar{g}\;\ldots \;g_{n}-\bar{g}\right]$ denote the zero-centered gene expression matrix, where $\bar{g}=\frac{1}{n}\sum_{i=1}^{n}g_{i}$. To decompose the co-expression matrix, we model it as a function of the largest components of variation. This is consistent with the assumption that the biology of a system can be explained by a subset of variance components among the $\left(\begin{array}{c} p\\ 2 \end{array}\right)$ pairwise gene-gene relationships. This allows us to preserve explainability, keeping all genes in the analysis within the context of a model in which functionally related groups of genes act together to define and alter biological states.

Our approach to identifying the relevant components is to find a set of eigenvectors of the sample co-expression matrix $C:=GG^{T}$ that best explains the gene co-variation, using the fact that in high-dimensional settings, where *p *>* n*, the rank of *C* will be r≤n, resulting in at most *n* nonzero eigenvalues. By keeping only the eigenvectors of *C* corresponding to nonzero eigenvalues, we can substantially reduce the parameter space from $O(p^{2})$ to O(n). We then consider the reduced eigendecomposition $C=\mathrm{QD}Q^{T}$, where **D** is a diagonal matrix for the nonzero eigenvalues of **C**, and the columns of **Q** correspond to their respective eigenvectors. We incorporate sample-specific covariates in the design matrix to infer a diagonal matrix of “pseudo-eigenvalues,” that represent the effect of each covariate on each nonzero eigenvalue. This reduces to solving the following optimization problem,

(1) $$\underset{\Psi }{\text{arg}\;\text{min}}||C-\frac{1}{n}\sum_{i=1}^{n}Qd\mathrm{iag}(X_{i}^{T}\Psi )Q^{T}|{|}_{F}^{2},$$

where Ψ is a *q *×* r* parameter matrix of coefficients that adjusts the eigenvalues as a function of the covariates to minimize the co-expression reconstruction error.

For example, in the case of a single batch and the absence of other experimental conditions ($X_{i}=1$ for all i∈[n]), then Ψ becomes identical to the vector of eigenvalues of the original covariance matrix. We show (Supplementary Text S1) that the optimization problem in Equation (1) is equivalent to solving *r* linear regression problems with the following closed-form solution:

(2) $${\hat{\Psi }}_{\cdot ,h}={\left(X^{T}X\right)}^{-1}X^{T}{\left[Q_{\cdot ,h}^{T}G_{\cdot ,i}G_{\cdot ,i}^{T}Q_{\cdot ,h}\right]}_{i=1}^{n}$$

for all h∈[r]. Moreover, by including an intercept in the first column of the design matrix **X**, we achieve a global minimum in Equation (1) and derive the following eigendecomposition:

$$\begin{array}{c} C=Q\mathrm{diag}(\frac{\sum_{i=1}^{n}X_{i}^{T}}{n}\hat{\Psi })Q^{T}\\ =\sum_{k=1}^{q}{\bar{X}}_{k}Q\mathrm{diag}({\hat{\Psi }}_{k,\cdot })Q^{T}\\ =Q\mathrm{diag}({\hat{\Psi }}_{1,\cdot })Q^{T}+\sum_{k=2}^{q}{\bar{X}}_{k}Q\mathrm{diag}({\hat{\Psi }}_{k,\cdot })Q^{T}\\ =:\bar{C}+\sum_{k=2}^{q}C_{k}, \end{array}$$

where ${\bar{X}}_{k}:=\frac{1}{n}\sum_{i=1}^{n}X_{i,k}$. COBRA decomposes the co-expression matrix as a linear combination of components, one for each covariate. It is worth noting that the experimental batch can be included among the covariates in **X**, allowing estimation of the batch effects as well as exploration of other covariates that induce patterns of gene co-expression.

## 3 Results

As a test of COBRA, we used simulated and real data to explore its use in case/control comparisons, batch effect removal, and extraction of covariate-specific patterns of co-expression.

### 3.1 Improved co-expression estimates *in silico*

To investigate the performance of COBRA in identifying covariate-specific co-expression, we simulated gene expression for 4000 genes in each of 400 samples, where each sample was assigned to a batch group (A or B) and a treatment group (case or control), mimicking a common experimental paradigm designed to avoid confounding treatment and batch. Based on the specific treatment-batch combination, we drew the gene expression levels for sample *i* from a multivariate Gaussian distribution with mean nine and covariance

$$\begin{array}{c} \Sigma_{i}:=\Sigma_{\text{background}}+\Sigma_{\text{case}}I_{\left[i\in \text{case}\right]}+\Sigma_{\text{control}}I_{\left[i\in \text{control}\right]}\\ +\Sigma_{\text{batch}\;A}I_{\left[i\in \text{batch}\;A\right]}+\Sigma_{\text{batch}\;B}I_{\left[i\in \text{batch}\;B\right]}, \end{array}$$

where the individuals components Σ_*j*_ for j∈{background, casecontrol, batch A, batch B} were sparse. This allowed us to reconstruct the ground truth: the nonzero pairs in $\Sigma_{\text{case}}-\Sigma_{\text{control}}$ represented “real” treatment effects, while the nonzero pairs in $\Sigma_{\text{batch}\;A}-\Sigma_{\text{batch}\;B}$ corresponded to “batch” effects.

To generate covariance components, we created 10 modules. The genes within each module were assigned a large absolute pairwise covariance, sampled uniformly from [−1.5,−0.5]∪[0.5,1.5], and selected independent of genes outside the particular module. This produced a covariance matrix *C_k_* for each module *M_k_* (k∈[10]) in which only a few entries, 1% in expectation, were nonzero.

We assigned each module to background ($M_{1},\ldots ,M_{5}$), case (*M*_6_ and *M*_7_), control (*M*_8_), batch A (*M*_9_), or batch B (*M*_10_), resulting in $\Sigma_{\text{background}}:=\sum_{k=1}^{5}C_{k},\;\Sigma_{\text{case}}:=C_{6}+C_{7},\;\Sigma_{\text{control}}:=C_{8},\;\Sigma_{\text{batch}\;A}:=C_{9}$, and $\Sigma_{\text{batch}\;B}:=C_{10}$. This simulation corresponds to a design matrix that has three variables representing three covariates: an intercept for the average co-expression, a batch (0 for Batch A, 1 for Batch B), and a treatment (1 for case, 0 for control). The resulting COBRA differential co-expression matrix represents the treatment component in the eigendecomposition.

We tested whether COBRA could discriminate treatment effects—the nonzero element in $\left(C_{\text{case}}-C_{\text{control}}\right)$—from all others. As baselines, we included the following batch correction methods: ComBat (Johnson *et al.* 2007), RUVCorr v1.32.0 (Freytag *et al.* 2015), SVA v3.48.0 (Leek *et al.* 2012), and LIMMA v3.56.2 (Ritchie *et al.* 2015). For each method, we corrected batch effects in gene expression, built separate co-expression matrices for case and control samples, and then computed the difference between these matrices. For comparison, we used a “naive method” in which we simply subtracted the case and control co-expression networks without correction. We also introduced a “naive batch” method for which we computed group differences within each batch and averaged these across batches:

$$\frac{1}{2}\sum_{b\in \{A,B\}}\left({\hat{C}}_{\text{case},\text{batch}\;b}-{\hat{C}}_{\text{control},\text{batch}\;b}\right),$$

where, ${\hat{C}}_{\text{case},\text{batch}\;A}$ is, for example, the Pearson correlation calculated for case samples in batch A.

**Figure 2.**
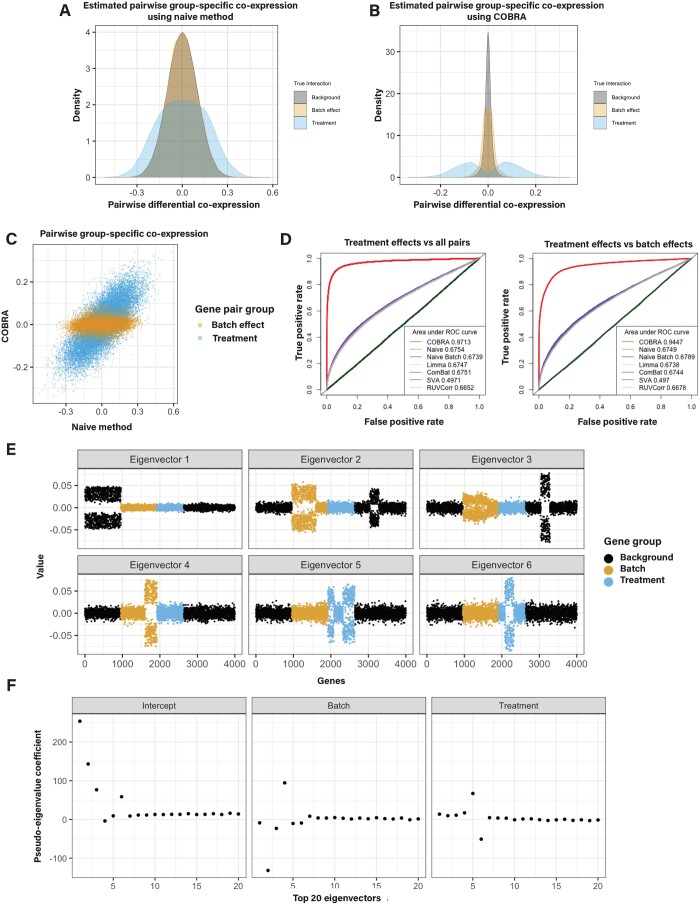
Simulation studies. (A) Naive differential co-expression scores and (B) COBRA scores for treatment, batch, and background effects. The Pearson correlation difference (the “naive” method) failed to discriminate true co-expression from background patterns and incorrectly identified batch effects as real treatment effects. COBRA identified treatment effects distinct from both background and batch effects. (C) Predicted scores of non-background gene pairs for naive differential co-expression (*x*-axis) versus COBRA (*y*-axis) demonstrate an improved separation of treatment effects (blue) from batch effects (orange). (D) ROC curves show the performance of COBRA and alternate methods in identifying treatment gene pairs compared to all pairs (left) and in comparison to batch genes (right). (E) Graphical representation of the top six eigenvectors of the original co-expression matrix plotted for 4000 simulated genes. Each point is colored according to each gene’s membership in batch, treatment (case or control), or background modules. Eigenvectors tend to separate along with co-expression modules. (F) COBRA-computed pseudo-eigenvalues for the top 20 eigenvectors corresponding to the three covariates (intercept, batch, treatment). Deviations from zero on the *y*-axis are indications of an unequal contribution of the corresponding eigenvector to the fitted co-expression estimate as shown in panel E; eigenvectors 5 and 6 have nonzero pseudo-eigenvalues corresponding to treatment effects.

For the naive method, we found that the co-expression values for treatment-induced gene pairs (case versus control) and batch-induced gene pairs were confounded (Fig. 2A). However, they are more clearly separated after COBRA correction (Fig. 2B), showing that COBRA can better discriminate “real” treatment effects from batch effects. When comparing COBRA and naive estimates for batch and treatment effects, we found that COBRA’s eigendecomposition effectively separates each covariate (Fig. 2C).

We computed receiver operating characteristic (ROC) curves comparing real effects (positive class) against all pairs (negative class) (Fig. 2D, left panel), as well as comparing real effects (positive class) and batch effects (negative class) (Fig. 2D, right panel). For the first case, COBRA outperforms other methods with an AUROC of 0.97, as compared to 0.68 for the runner-up naive method. For the second case, COBRA’s AUROC was 0.94, while the naive batch achieved only 0.68. Runtimes for each are reported in Supplementary Table S2.

We adapted the data-generating mechanism described above to simulate using COBRA to extract covariate-related co-expression among genes in samples derived from a single experimental condition but two batches (Supplementary Text S3). We found that in this situation, COBRA was able to identify and remove batch effects from gene co-expression (Supplementary Fig. S4). This was further verified by another simulation experiment in which we used lung gene expression data and added artificially induced residual batch effects (Supplementary Text S2).

To demonstrate the interpretability of COBRA’s estimates, we performed an eigendecomposition of the original co-expression matrix. We plotted the top six eigenvectors for all genes (Fig. 2E) and found that the eigenvectors tend to separate along with co-expression modules. Then, we computed COBRA’s pseudo-eigenvalues for the top 20 eigenvectors corresponding to the three covariates. COBRA assigned the largest absolute pseudo-eigenvalue coefficient (Fig. 2F) to the correct module in the original co-expression matrix (Fig. 2E). For example, the coefficient vector corresponding to treatment has large absolute eigenvalues for components 5 and 6 (Fig. 2F), which correspond to real treatment effect gene pairs (Fig. 2E). In this case, the estimate $\hat{\Psi }$ is a 3 × 4000 matrix, in which the value in the *ith* column and *jth* row can be interpreted as the additional contribution of the *ith* eigenvector for a one unit increase in the value of the *jth* variable.

### 3.2 Correction of a controlled introduction of batch effects in ENCODE

We benchmarked COBRA’s effectiveness in removing non-biological differential co-expression using human gene expression data consisting of 153 RNA-Seq profiles for 12 424 genes in lymphoblastoid cell lines from the ENCODE project (Pickrell *et al.* 2010). Using the pre-processing steps described by Kuijjer *et al.* (2019), we used only data from the 63 individuals who had been sequenced both at Yale University and at Argonne National Laboratory (126 RNA-Seq profiles). Because both centers used the same sequencing instrument (Illumina Genome Analyzer II), we considered the centers to represent two independent batches for correction.

**Figure 3.**
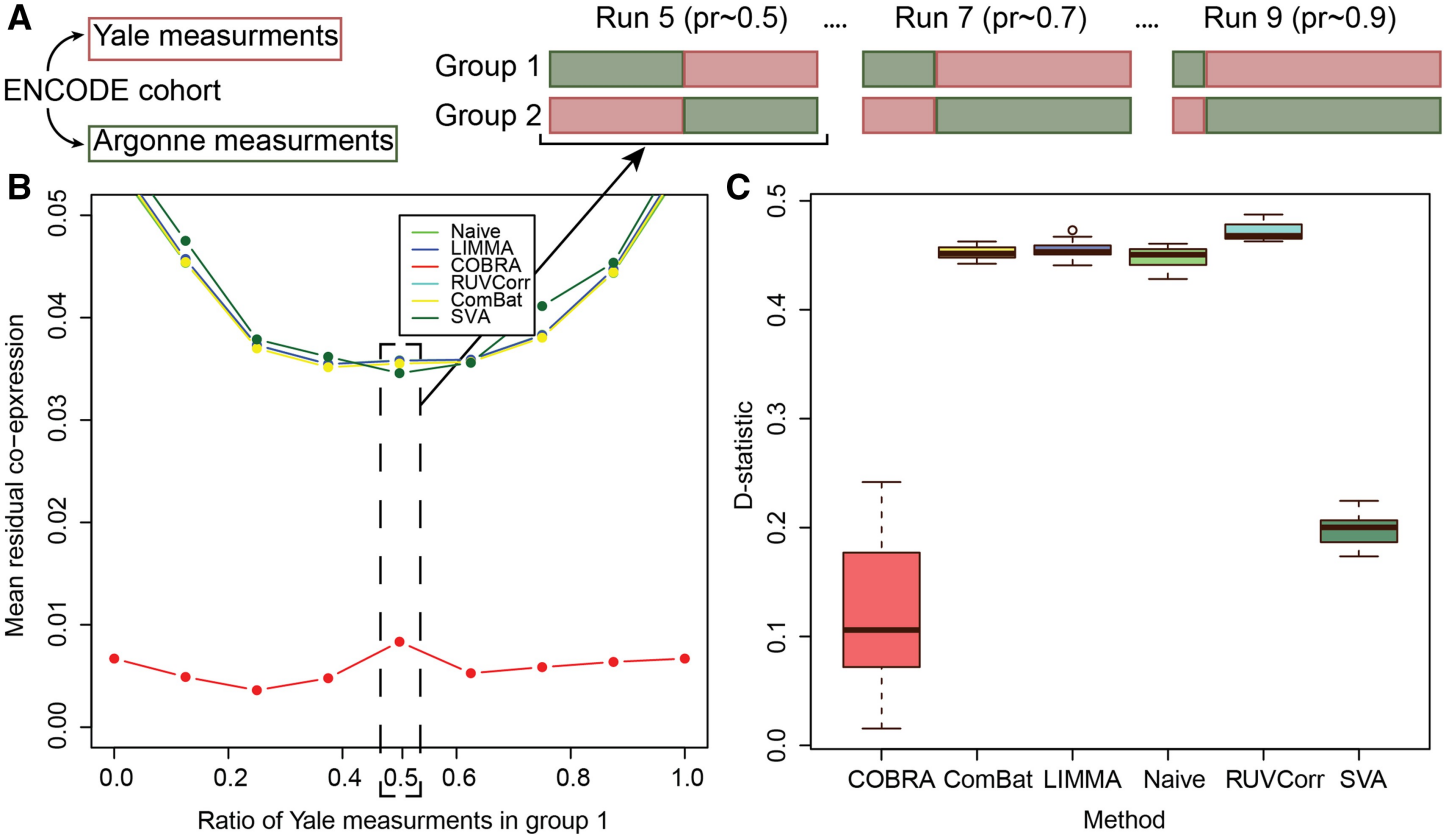
Comparison of residual co-expression between centers. (A) Design of the controlled batch effect experiment. Gene expression data were generated for all participants at both Yale and Argonne, so the “center” is equivalent to a batch variable. We created two groups RNA-Seq profiles in which each individual was sampled only once, varying the fraction of samples measured at each center. Sample sets with ∼50% of samples from each center were referred to as “balanced” and all others as “unbalanced.” (B) Residual co-expression. The residual co-expression (*y*-axis) for different proportions (*x*-axis) of Yale and Argonne measurements in group 1 and group 2; COBRA reduced the residuals at least by a factor of four. (C) Stability of unbalanced design (pr = 0.25) versus balanced design, showing D-statistics for the null hypothesis of the equality of residual co-expression’s empirical distributions for the unbalanced (pr = 0.25) and the balanced (pr = 0.5) designs using COBRA, ComBat, LIMMA, naive, RUVCorr, and SVA.

We constructed mixed test data sets, each containing differing proportions of data from each of the two laboratories (Yale and Argonne) in fractional steps ranging from 0% to 100%; we referred to the sample sets that contained ∼50% of their samples from each group as “balanced,” and all others as “unbalanced” (Fig. 3A). We repeated each of these mixing experiments ten times and averaged the results. For each analysis, the design matrix included an intercept to represent average co-expression, batch (Yale or Argonne), and a binary group variable.

After location-scale batch correction, we performed between-group differential co-expression analysis. Because the only differences between groups were the centers at which the RNA-seq data was generated, one might expect not to see group-specific co-expression. However, when we calculated the mean residual co-expression across our ten replicates at each mixing proportion, we found a significant artifactual co-expression between genes. When we used COBRA, that residual co-expression was reduced by at least a factor of four (Fig. 3B), illustrating that COBRA can effectively estimate and eliminate batch-dependent co-expression.

We assessed the robustness of COBRA’s estimates between the balanced and unbalanced designs by selecting the 1000 most variable genes between lab batch “groups” and computing the Kolmogorov–Smirnov D-statistic,

$$D:=\underset{x}{\sup}|F_{1}(x)-F_{2}(x)|,$$

where *F*_1_ and *F*_2_ are the empirical cumulative distributions for the balanced and unbalanced designs, respectively; these were estimated by resampling the data 1000 times to build different partitions. We tested for the equality of the empirical distributions of the residual group-specific co-expression using the Kolmogorov-Smirnov test, computing ten estimates of the D-statistic (Fig. 3C); for the D-statistic a smaller value corresponds to weaker evidence against the null hypothesis of the distributions being equal. In our analysis, we found that COBRA has consistently smaller D-statistics, a measure of its greater robustness.

### 3.3 Analysis of cancer-specific co-expression modules in thyroid cancer

We downloaded TCGA human thyroid carcinoma (THCA) RNA-Seq data (Agrawal *et al.* 2014) for 572 individuals from recount3 (Wilks *et al.* 2021) with sequences mapped to the GENCODE v26 reference gene set. We considered only protein-coding genes and removed genes without reads in any sample, leaving 19 711 genes. We used *transform_counts* from the recount3 package (Wilks *et al.* 2021) to scale the RNA-Seq data and then normalized it using Variance Stabilizing Transformation from DESeq2 (Love *et al.* 2014). Using the reported metadata, we selected six covariates: sex (415 females, 157 males), race (381 White, 104 not reported, 54 Asian, 32 Black, 1 American Indian), stage (325 stage I, 59 stage II, 125 stage III, 2 stage IV, 51 stage IVa, 8 stage IVc, 2 not reported), batch (17 batches), age (as a continuous variable; mean 47, min 15, max 89), and case/control status [the 513 cancer samples, metastatic and primary tumor, were classified as case instances (tumor); the 59 normal tissue adjacent to tumors (NAT) samples as controls]. We encoded all categorical variables using *dummy_columns* from fastDummies v 1.7.3.

**Figure 4.**
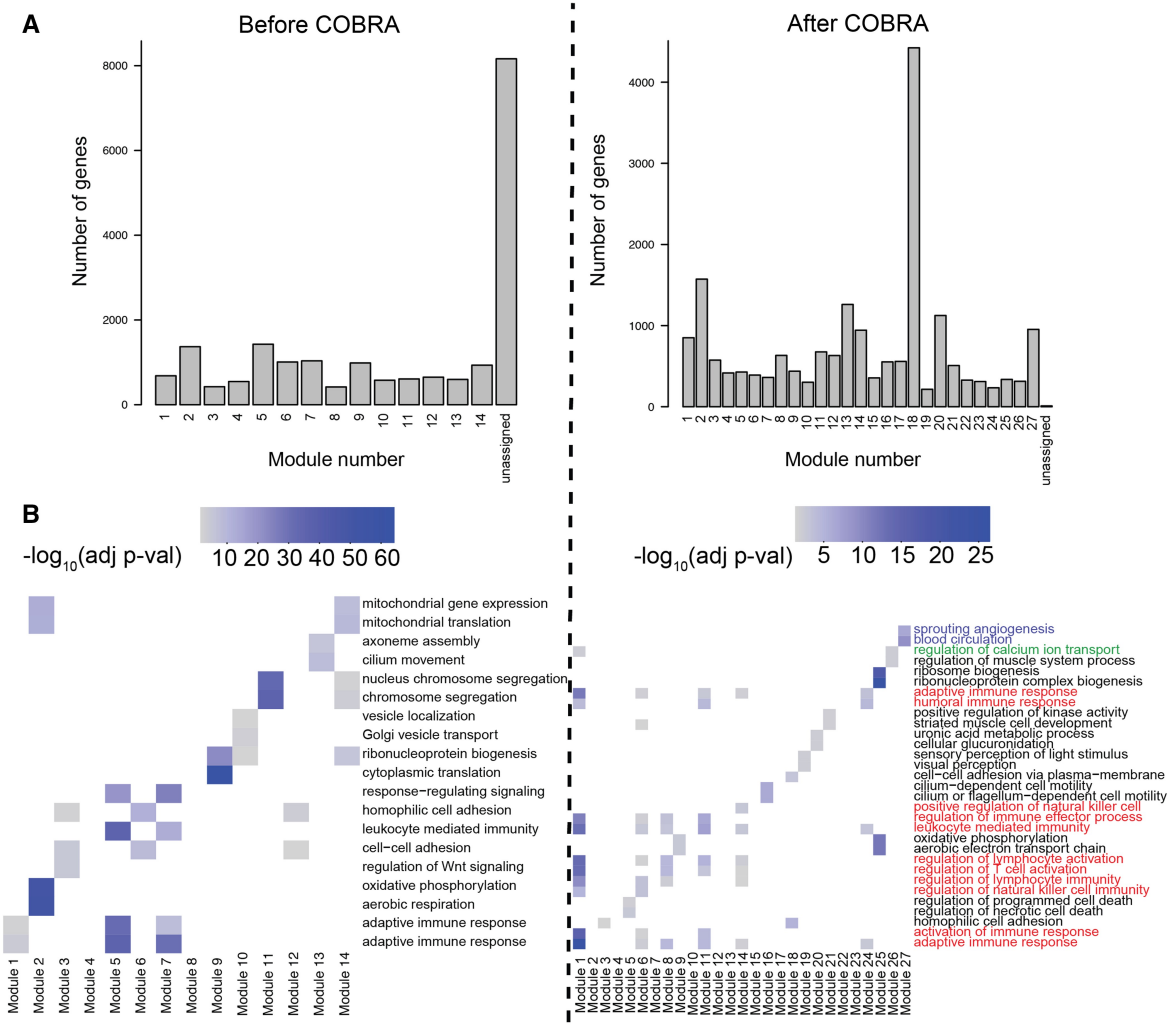
Functional modules in COBRA-corrected THCA co-expression networks. (A) Size of functional modules in co-expression networks as computed using WGCNA before and after COBRA correction. (B) Gene set enrichment analysis of functional modules based on Gene Ontology (GO) biological process terms before and after COBRA correction. Heatmap color intensity is associated with the significance of GO terms; colored labels represent different clusters described in the text.

We computed cancer-specific co-expression networks in two ways. First, we subtracted the Pearson correlations for cancer and NAT samples. We took the absolute value of each correlation “edge” to produce a “naive network” in which large values correspond to large differences between cancer and healthy samples. Note that this approach corresponds to the absolute value of the naive method we introduced in the *in silico* network analysis. Next, we ran COBRA with a design matrix containing intercept, binary variable for cancer (0 for NAT, 1 for cancer), sex (0 for male, 1 for female), age, race, stage, and batch; all categorical variables were coded as binary variables. After COBRA eigendecomposition, we extracted the co-expression component corresponding to the cancer variable and took its absolute value, with the whole procedure taking around seven minutes on a r5.2xlarge instance (Supplementary Table S3). Since we used aggregated cancer and NAT data as input to COBRA, the eigendecomposition decouples cancer effects from batch and other covariates, producing a cancer-specific gene co-expression network. For both naive and COBRA co-expression networks, we used WGCNA (Langfelder and Horvath 2008) to identify modules of correlated genes (Fig. 4A).

WGCNA begins with a pairwise correlation matrix that it transforms into an adjacency matrix by raising each correlation to a “soft thresholding power” determined using a scale-free topology criterion. WGCNA creates a topological overlap matrix (TOM) that measures the similarity in the co-expression patterns of genes, taking into account shared neighbors in the network. Average linkage hierarchical clustering is then performed, and the resulting dendrogram is cut to identify modules; we imposed a minimum cluster size of 200 genes. We performed functional enrichment analysis using Gene Ontology (GO) biological process terms (Fig. 4B) and Kyoto Encyclopedia of Genes and Genomes (KEGG) pathway terms (Supplementary Fig. S2) using *enrichGO* and *enrichKEGG* from clusterProfiler v4.10 (Wu *et al.* 2021) (one-sided Fisher’s exact test, *P *<* *.05 after Benjamini–Hochberg FDR correction).

We found that COBRA-corrected WGCNA networks had both a more complex community structure (27 modules) than the naive method networks (14 modules), and fewer unassigned genes (Fig. 4A). In the cluster annotation of the two most significant terms for each module (Fig. 4B), COBRA was able to resolve the clustering of similar functional gene classes into more precisely defined functional groups by removing residual batch effects. Of the 27 COBRA modules, 17 matched at least one significant GO annotation and 12 matched a KEGG term. Module 1 corresponds to cancer immune response pathways (T-cell lymphocytes and natural killer cell recruitment; red annotation). Module 8 contains co-expressed genes involved in the regulation of calcium transport, which is an important physiological role of thyroid glands mediated by hormone secretion (green annotation). Module 27 includes genes associated with angiogenesis and blood circulation, both of which are important for tumor development and overall disease severity (blue annotation). KEGG modules (Supplementary Fig. S2) added additional evidence supporting the involvement of inflammatory processes (cytokines) for Module 1 and confirmed disrupted calcium signaling in module 27. Module 14 includes genes over-represented for osteoclast differentiation, which is known to be induced by thyroid hormones. As a validation, we generated five datasets using bootstrapping with replacement and consistently found the same pathways reported above (Supplementary Figs S5 and S6). In contrast, functional enrichment analysis of the modules from the naive networks identified enrichment of fewer meaningful processes and included spurious terms such as COVID-19 and cardiomyopathy (Supplementary Fig. S2), indicating that there may be some residual batch-dependent correlation or other artifacts in the data.

As an additional objective measure of COBRA’s performance, we used the strategy developed by Skinnider *et al.* (2019) and ran EGAD (Ballouz *et al.* 2017) using GO biological process terms as the annotation source. We found that COBRA corrections for sex, age, stage, race, and batch improved the EGAD AUROC for Pearson correlation from 0.647 to 0.650. This change, though minor, demonstrates the value of removing spurious batch-dependent correlation.

**Figure 5.**
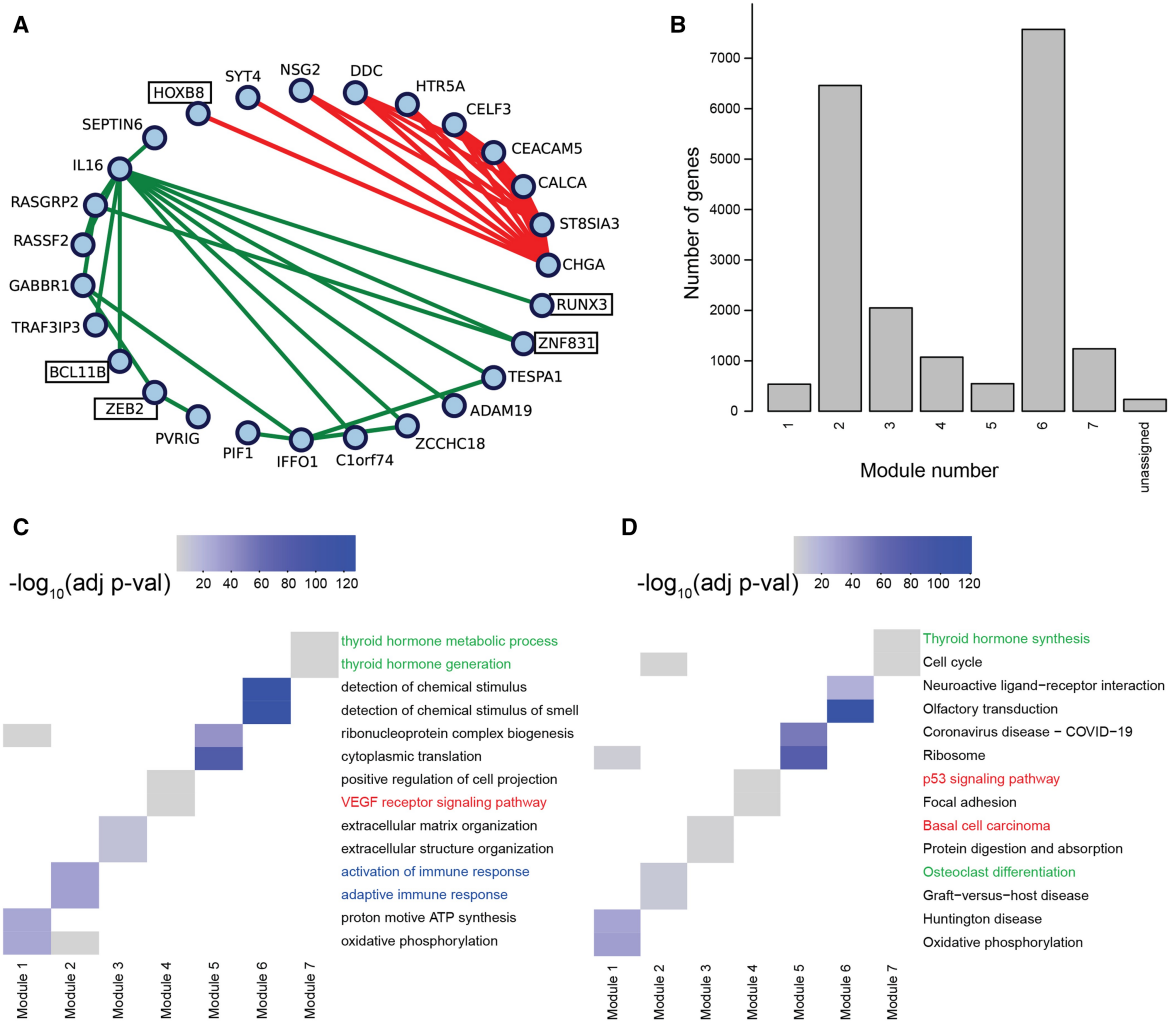
Functional modules in COBRA-corrected partial correlation THCA networks. (A) Largest 20 positive and negative edge weights. Green lines (lower fully connected subgraph) indicate positive partial correlations, red lines (upper fully connected subgraph) indicate negative partial correlations. (B) Functional modules from WGCNA’s module detection on COBRA-corrected partial correlation networks. (C) Gene set enrichment analysis of functional modules using GOBP and (D) KEGG annotations. Colored labels represent different clusters described in the text.

In addition to Pearson correlation, COBRA can be used with other measurements of gene–gene association. Partial correlations, in particular, have been shown to reduce the number of spurious associations in high-dimensional co-expression networks (Shutta *et al.* 2023), so we used the *pcor.shrink* function from the corpcor package (Schafer *et al.* 2017) to estimate partial correlation networks in the THCA RNA-Seq data. We decomposed the resulting network using COBRA, correcting for batch and the other covariates and extracted the component corresponding to the cancer. We examined gene pairs with the 20 largest positive and negative edge scores, which included interactions between 27 genes (Fig. 5A). Among these were immune process genes such as IL16, which mediates macrophage polarization, and HTR5A, which enhances innate immunity, as well as genes such as calcitonin-related polypeptide alpha (CALCA) that encodes the release of calcitonin, a hormone secreted by the thyroid gland that plays a role in calcium homeostasis.

We used WGCNA’s module detection on COBRA-corrected partial correlation networks, which mapped nearly all genes to one of seven modules; we performed GSEA on these modules and selected the two most significant terms for each module (Fig. 5B). In GO Biological Process (GOBP) analysis, we found significant terms related to the activation of immune response in Module 2 (blue annotation in Fig. 5C), cancer-related signaling pathways (VEGF and p53 signaling) in Module 4 (red annotation), and thyroid-associated functions in Module 7 (green annotation). Using KEGG (Fig. 5D), we found terms related to thyroid physiology, such as hormone synthesis (Module 7) and osteoclast differentiation (Module 4).

### 3.4 Building accurate gene regulatory networks using COBRA batch-corrected co-expression

In our THCA partial correlation networks (Fig. 5A), there are a number of transcription factors (TFs) that map to co-expression modules, including BCL11B, HOXB8, RUNX3, ZEB2, and ZNF831, all of which have been reported as linked to THCA; their inclusion in the partial correlation modules suggest that these TFs may regulate the expression of their correlated partners.

Because many gene regulatory network (GRN) inference methods use co-expression as an input, we investigated how COBRA batch correction affects GRN inference. We computed a COBRA covariate-specific co-expression network for cancer using cancer cases and NAT as controls, adjusting for sex, race, stage, batch, and age. We used this COBRA-corrected co-expression matrix as input to PANDA (Glass *et al.* 2013). In addition to a co-expression matrix which serves as evidence of co-regulation, PANDA takes two additional input prior networks: a transcription factor protein-protein interaction network [PPI; downloaded from the GRAND database v1.5.4 (Ben Guebila *et al.* 2022a)] which captures the fact that some TFs work coordinately to regulate genes, and a “network prior” GRN (based on mapping TF binding motifs to the genome).

In the resulting GRN, the weight of each edge quantifies the relative amount of evidence supporting the existence of a TF-gene regulation relationship. Among the TFs with the greatest edge scores are members of the CEBP (CCAAT/enhancer-binding protein) and POU (Pit-Oct-Unc) TF families (Supplementary Fig. S3A). The POU domain TF family is interesting in the context of cancer as it includes key TFs linked to developmental processes that include embryonic pluripotency and neuronal specification. The POU TFs have not generally been associated with THCA. However, OCT1/POU2F1 is known to be highly expressed in THCA and it regulates genes associated with proliferation, immune modulation, and oxidative and cytotoxic stress resistance. OCT1 is known to be pro-oncogenic in multiple contexts and has prognostic and therapeutic value in various epithelial tumors, including THCA (Vázquez-Arreguín and Tantin 2016). The TF SOX12, which shows increased expression in THCA tissue and cells compared to normal thyroid, promotes cell proliferation, migration, invasion, and tumor growth and has been reported to interact with members of the POU family. SOX12 knockdown inhibits expression of POU2F1 and POU3F1; expression of POU2F1 and POU3F1 has been shown to reverse the effects of SOX12 knockdown in THCA cells, indicating their role in its progression (Su *et al.* 2022).

We then took a gene-centric approach and calculated a “gene targeting score,” defined as the weighted sum of its TF-gene in-degree in the PANDA GRN, and performed gene set enrichment analysis on those genes with high and low targeting scores. As shown in Supplementary Fig. S3B and C, based on OMIM annotation, these genes are enriched for genetic signatures of thyroid cancer and other malignancies, demonstrating that the COBRA-corrected correlation matrices as input to PANDA GRN estimation provide meaningful biological insight into disease processes.

## 4 Discussion

The correction of batch effects has long been recognized as essential for meaningful and accurate analysis of gene expression patterns, and there are now a number of batch correction methods that have been shown to dramatically improve the fidelity of between-group comparisons. However, these methods have only addressed batch effects in the mean and variance of individual genes, ignoring higher-order effects that might induce spurious, batch-dependent correlations between genes. Identifying and removing these spurious, non-biological correlations is important as phenotypic changes are driven by alterations in specific biological functions and pathways; changes that manifest themselves through coordinated co-expression of related genes or in regulatory networks consisting of transcription factors and the genes they control (Glass *et al.* 2013, Kuijjer *et al.* 2019). COBRA addresses the gap in batch correction methods by introducing a scalable matrix-factorization approach that identifies components of the co-expression matrix corresponding to the sample batch and other covariates. These components can subsequently be subtracted out or used in further analyses exploring the effects of a particular covariate on patterns of co-expression.

Our simulation studies demonstrated that COBRA was more effective in removing batch-dependent correlation patterns than approaches that performed standard first-order batch correction followed by calculation of correlation matrices. We also showed that the performance of COBRA was stable as the relative proportion of samples in each batch varies—an important consideration when analyzing real-world data in which one often has little control of where samples are collected or data is generated. To verify these results, using ENCODE RNA-Seq data generated on the same samples at two different sequencing centers, we explored the behavior of COBRA under various batch mixing scenarios and found that COBRA performed far better than other methods.

The real test of any method is how well it performs on “real world” data. Using data from thyroid carcinoma (THCA) in TCGA, we showed that COBRA has advantages over the naive first-order methods. Using WGCNA to estimate correlation networks, we found a richer, more biologically nuanced co-expression structure whose modules were better linked to THCA biology than did the use of standard mean/variance normalization methods before WGCNA analysis; these results were validated in a bootstrap analysis and functional enrichment analysis using EGAD.

We also used COBRA to infer partial correlation networks between TFs and genes from which we took the 20 highest-scoring positively and negatively correlated edges, among which were seven TF-gene associations involving five transcription factors (TFs). Given that TFs represent only a small fraction (<10%) of protein-coding genes, this is far more than we would expect by chance and suggests that these TFs may play a key role in THCA development and progression. Using the same data, we demonstrated that COBRA co-expression matrices could be effectively used to estimate and compare GRNs between conditions and identified transcription factors that may play a substantial role in THCA—identifications not possible using standard normalization methods alone. At the same time, we showed that the computational time and resources required by COBRA were not that much greater than what is required by naive methods. All of this argues for the use of COBRA whenever performing any analysis that relies on unbiased estimates of gene-gene correlation patterns based on expression data.

The reason for the improved performance of COBRA has to do with the assumptions that are built into any batch correction approach. Previous methods to which we compared COBRA are broadly “first-order” in the sense that they use various techniques to adjust the mean and variance of the distribution of expression values such that these are consistent between batches. When we used these batch-corrected values to calculate correlation-based co-expression networks, we found that they retained residual batch-dependent correlations. COBRA represents a conceptually different approach to batch correction. COBRA is the first method that corrects for higher-order batch effects by decomposing the starting co-expression correlation matrix into a linear combination of covariate-associated co-expression matrices, thus explicitly addressing biases in correlation patterns between genes.

Although COBRA has significant advantages, it has one potential drawback. Correlation coefficients, by definition, are constrained to lie in the [−1,1] interval, but the entries of the matrices calculated by COBRA can fall outside that interval. Although potentially problematic, in practice, we have not seen this to be a significant problem. In the THCA analysis we presented, at most, 5.9% of COBRA co-expression matrix entries fell outside the expected range (Supplementary Table S1). This could potentially be addressed by normalizing COBRA’s batch-corrected covariance matrix to constrain values to [−1,1], although the best way to do this is not immediately obvious.

COBRA represents a significant step forward in batch correction methodology, allowing more effective removal of batch-dependent correlation structures and providing a way of identifying covariate-specific patterns of co-expression in gene expression data. This ability to remove spurious correlations is essential for improving the fidelity and interpretability of correlation-based and gene regulatory networks. Since beginning the analysis described in this article, we have extended COBRA to use DRAGON’s multi-omic partial correlation networks (Shutta *et al.* 2023) and plan to implement it for other methods as well.

Finally, we should note that although we have focused on correlations in gene expression, COBRA is a general method and not limited in its application. One could easily use COBRA for other correlation-based measures of association and in other application areas. One potentially interesting application would be to metagenomic classification, in which correlations between microbial species are used to distinguish between states observed in microbial ecology and human health.

## Supplementary Material

btae531_Supplementary_Data

## Data Availability

COBRA is available under the GLP3 open source license in R and Python in netZoo (Ben Guebila *et al.* 2023) (https://netzoo.github.io) with tutorials in Netbooks (https://netbooks.networkmedicine.org) (Ben Guebila *et al.* 2022b). The code and data for the analysis are in GitHub (https://github.com/QuackenbushLab/cobra-experiments). Human RNA-Seq data from ENCODE were downloaded from GEO (GSE19480). Processed input data have been deposited in Zenodo (https://zenodo.org/records/10154758).
