# Supplementary material for: Higher-order correction of persistent batch effects in correlation networks
Source: Bioinformatics. 2024 Sep 3;40(9):btae531. doi: 10.1093/bioinformatics/btae531 (PMC11441315; doi:10.1093/bioinformatics/btae531)
Supplement: btae531_Supplementary_Data [file btae531_supplementary_data.pdf]

Supplementary material for: Higher-order correction of persistent batch effects in correlation networks

Micheletti, Schlauch et al.

Contents

1 Optimization **1**

2 Batch co-expression is not corrected by standard correction methods **2**

3 COBRA can effectively identify and remove batch effects in a single co-expression matrix **5**

References **11**

1 Optimization

COBRA optimizes the following objective function (Equation 1 in the main text):

$$\arg\min_{\boldsymbol{\Psi}} ||\boldsymbol{C} - \frac{1}{n}\sum_{i=1}^{n} \boldsymbol{Q} diag(\boldsymbol{X}_i^T \boldsymbol{\Psi})\boldsymbol{Q}^T||_F^2, \tag{1}$$

where $\boldsymbol{C}$ is the sample covariance matrix of a zero-centered gene expression $\boldsymbol{G}$, and $\boldsymbol{Q}$ is the matrix of the eigenvectors corresponding to non-zero eigenvalues in the eigendecomposition $\boldsymbol{C} = \boldsymbol{Q}\boldsymbol{D}\boldsymbol{Q}^T$. The goal is estimating the matrix $\boldsymbol{\Psi} \in \mathbb{R}^{k \times r}$ (r being the number of non-zero eigenvalues of $\boldsymbol{C}$), quantifying the effect of the q covariates included in the design matrix on each eigenvalue. We now show that a global optimum for Equation 1 can be computed in closed form.

The first crucial observation is that since $\boldsymbol{C}$ is symmetric, $\boldsymbol{Q}$ is an orthogonal matrix. We get

$$\begin{aligned}
&\arg\min_{\boldsymbol{\Psi}} ||\boldsymbol{C} - \frac{1}{n}\sum_{i=1}^{n} \boldsymbol{Q} diag(\boldsymbol{X}_i^T \boldsymbol{\Psi})\boldsymbol{Q}^T||_F^2 \\
&= \arg\min_{\boldsymbol{\Psi}} ||\frac{1}{n}\sum_{i=1}^{n} \boldsymbol{G}_{\cdot,i}\boldsymbol{G}_{\cdot,i}^T - \frac{1}{n}\sum_{i=1}^{n} \boldsymbol{Q} diag(\boldsymbol{X}_i^T \boldsymbol{\Psi})\boldsymbol{Q}^T||_F^2 && \text{By definition} \\
&= \arg\min_{\boldsymbol{\Psi}} ||\frac{1}{n}\sum_{i=1}^{n} \left(\boldsymbol{G}_{\cdot,i}\boldsymbol{G}_{\cdot,i}^T - \boldsymbol{Q} diag(\boldsymbol{X}_i^T \boldsymbol{\Psi})\boldsymbol{Q}^T\right)||_F^2 && \text{Commutativity} \\
&= \arg\min_{\boldsymbol{\Psi}} ||\frac{1}{n}\sum_{i=1}^{n} \left(\boldsymbol{Q}^T\boldsymbol{G}_{\cdot,i}\boldsymbol{G}_{\cdot,i}^T\boldsymbol{Q} - diag(\boldsymbol{X}_i^T \boldsymbol{\Psi})\right)||_F^2 && \text{Orthogonal matrices preserve the Frobenius norm} \\
&= \arg\min_{\boldsymbol{\Psi}} \sum_{h=1}^{r} \left(\frac{1}{n}\sum_{i=1}^{n} \left(\boldsymbol{Q}_{\cdot,h}^T\boldsymbol{G}_{\cdot,i}\boldsymbol{G}_{\cdot,i}^T\boldsymbol{Q}_{\cdot,h} - \boldsymbol{X}_i^T\boldsymbol{\Psi}_{\cdot,h}\right)\right)^2 && \text{Optimizing } \Psi \text{ only affects diagonal elements}
\end{aligned}$$

Because the terms above are positive and each one corresponds to a different column of $\hat{\boldsymbol{\Psi}}$, we can decouple the optimization and solve for each $h \in [r]$ separately:

$$\hat{\boldsymbol{\Psi}}_{\cdot,h} = \arg\min_{\boldsymbol{\Psi}} \left(\sum_{i=1}^{n} \boldsymbol{Q}_{\cdot,h}^T \boldsymbol{G}_{\cdot,i} \boldsymbol{G}_{\cdot,i}^T \boldsymbol{Q}_{\cdot,h} - \boldsymbol{X}_i^T \boldsymbol{\Psi}_{\cdot,h} \right)^2 \quad (2)$$

In other words, we want to minimize the sum of the residuals for a standard linear regression. Here, every unbiased estimator achieves the optimum in Equation 1, and we chose the maximum-likelihood estimator (MLE) because was the candidate solution with the lowest variance. Assuming that the design matrix has an intercept column, the MLE has a closed-form solution:

$$\hat{\boldsymbol{\Psi}}_{\cdot,h} = (\boldsymbol{X}^T\boldsymbol{X})^{-1}\boldsymbol{X}^T \left[\boldsymbol{Q}_{\cdot,h}^T \boldsymbol{G}_{\cdot,i} \boldsymbol{G}_{\cdot,i}^T \boldsymbol{Q}_{\cdot,h} \right]_{i=1}^{n}. \quad (3)$$

2 Batch co-expression is not corrected by standard correction methods

To illustrate the presence of residual batch effects in co-expression data after standard batch effect correction, we created an artificial dataset using lung gene expression data from GTEx v7.0 [GTEx-Consortium, 2015] as processed and available in the GRAND [Ben Guebila et al., 2022] database v1.5.3. By sampling this data without replacement, we created two subsets to represent different batches. We created a first "batch" by randomly selecting 100 samples and, for those samples, 1000 genes and their expression levels (ignoring their identities and labeling them gene 1 through gene 1000). We then created a second "batch" by selecting another 100 random samples, but for these, we chose the expression levels of a different random set of 1000 genes to create a clear batch-dependent bias. The result was a gene expression matrix with 200 samples and 1000 genes but with distinct subsets based on the expression of their genes. As expected based on this construction, there is significant differential expression between these batches (Figure S1-A). As expected, when we corrected for group membership using ComBat, this differential expression completely disappeared (Figure S1-A).

We calculated differential co-expression and plotted the distribution of differential co-expression estimates between batches, which we refer to as differential co-expression "across batches" (Figure S1-B). We repeated this analysis, correcting for batch using either ComBat or LIMMA, and we also corrected for batch co-expression using COBRA. For comparison in each group, we calculated a differential co-expression "null distribution" by randomly selecting subsets of samples from the original matrix to remove the batch distinction and create "groups." We repeated this process ten times and averaged the outcome to obtain a "mean random" differential co-expression. Henceforth, we distinguish between groups and batches as the groups are sampled from the batches but, by construction, no longer have a clearly defined batch structure and so we expect neither differential expression nor differential co-expression.

In the subpanels of Figure S1-B, we plotted the distribution of uncorrected co-expression values, those following ComBat or LIMMA batch correction, and those corrected by COBRA, together with the relevant null distribution. As can be seen, the COBRA-corrected data almost perfectly matches the associated null distribution, demonstrating that the batches, after correction, become indistinguishable. In the other panels, there is a significant density of values above the null as the absolute co-expression increases. Together, these plots illustrate the overall efficiency of COBRA's removal of batch effects and the failure of other batch corrections to do so.

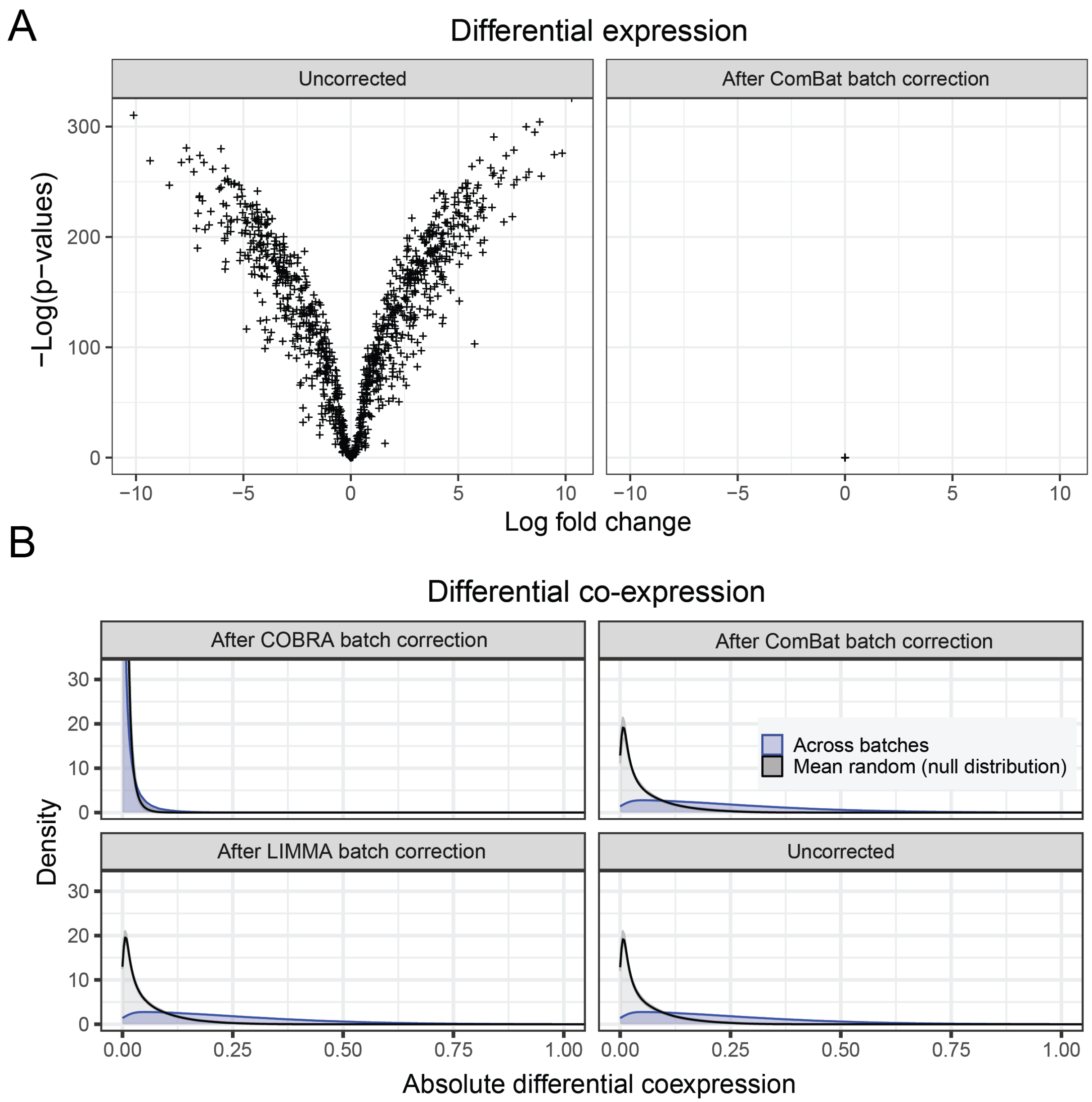

Figure S1: **Persistent batch effects in differential co-expression after standard batch correction. A. ComBat can correct differential expression between batches**, but differential co-expression persists even after correction. **B. Differential co-expression between groups using ComBat, LIMMA, and COBRA.** As can be seen, COBRA removes batch effects from co-expression, producing a distribution that approximates the null. In contrast, there is differential co-expression in the uncorrected data that persists even after ComBat or LIMMA adjustment such that there is an excess density of co-expression values relative to the null.

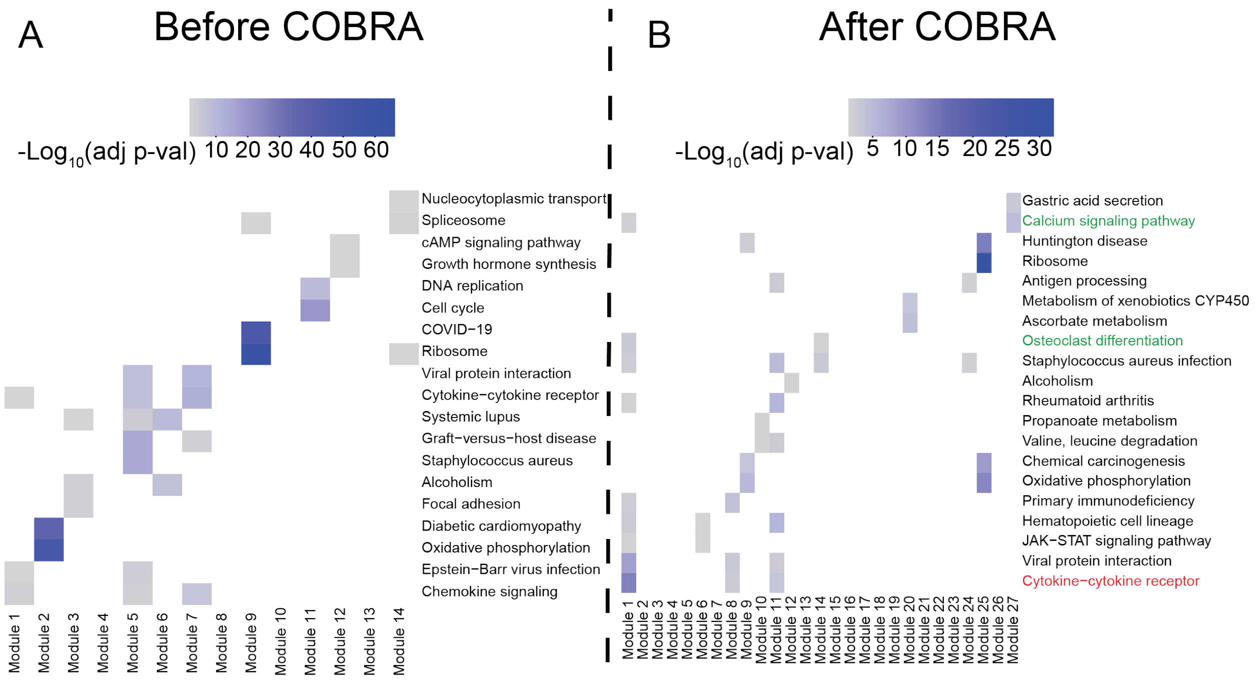

Figure S2: **Gene set enrichment analysis in KEGG database of thyroid cancer co-expression network.** Analyses were done **A. before COBRA correction** and **B. after COBRA correction** of gene co-expression. Color intensity is associated with significance levels of KEGG term enrichment.

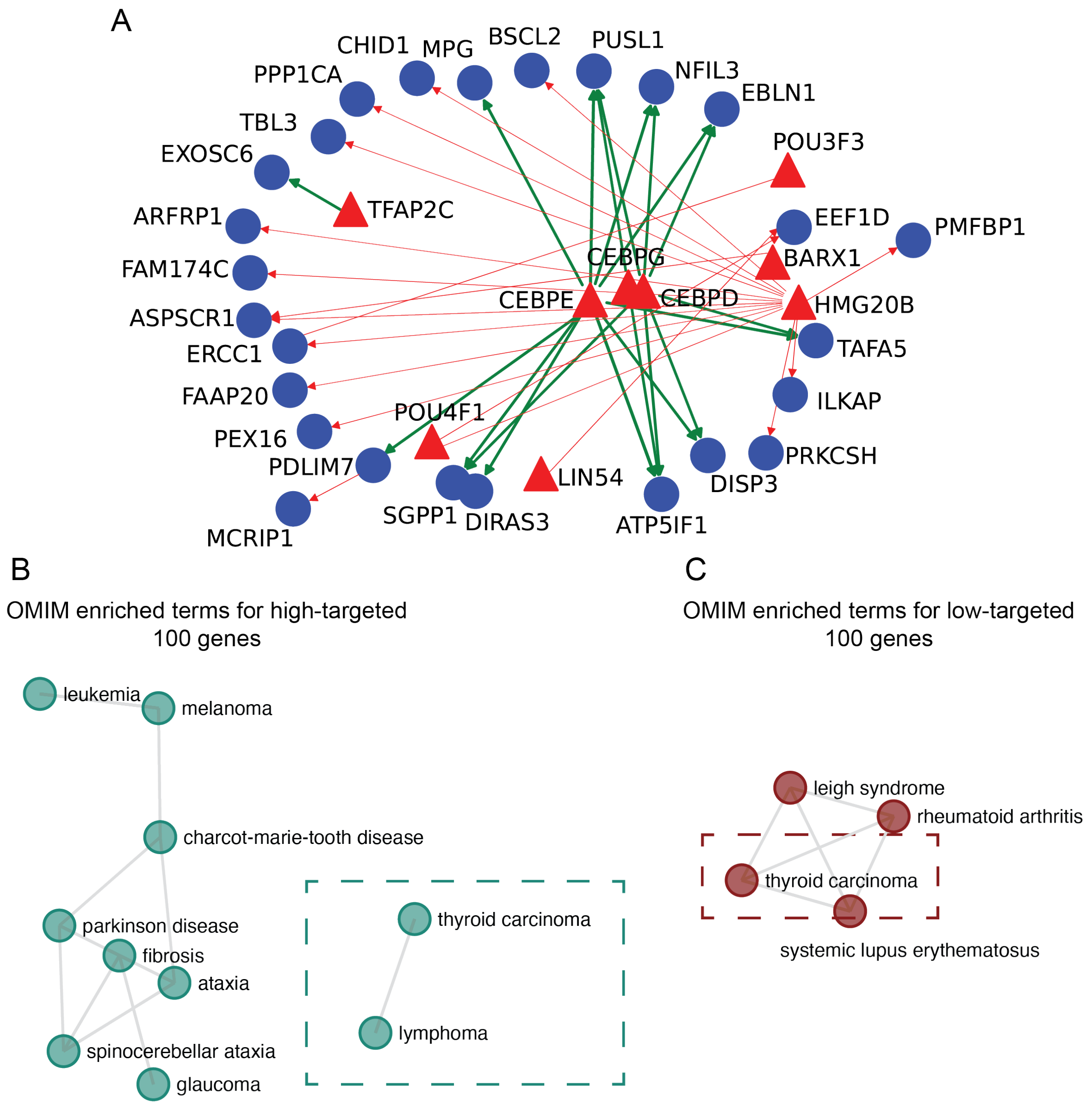

Figure S3: **Gene regulatory network inference in thyroid cancer using COBRA-corrected gene co-expression. A.** PANDA gene regulatory network inferred using COBRA co-expression data as input. The network represents the top 20 edge positive (green) and negative (red) edge weights. Red triangles represent transcription factors, and blue circles represent target genes. **B.** Gene set enrichment analysis of top 100 high-targeted and **C.** low-targeted genes in PANDA thyroid co-expression network in OMIM database.

3 COBRA can effectively identify and remove batch effects in a single co-expression matrix

To assess the performance of COBRA in identifying and removing batch effects from a single co-expression matrix, we conducted further investigations by slightly modifying the data-generating mechanism described for the *in silico* experiments presented in the main text. We simulated gene expression for 4000 genes in each of 400 samples, where each sample was assigned to a batch group (A or B). We drew the gene expression

levels for sample i from a multivariate Gaussian distribution with covariance Σ_i defined as:

$$\Sigma_i := \Sigma_{\text{batch A}} I_{[i \in \text{batch A}]} + \Sigma_{\text{batch B}} I_{[i \in \text{batch B}]},$$

where the individual components Σ_j for $j \in \{\text{batch A, batch B}\}$ were sparse. We created 10 modules with pairs of genes exhibiting a large absolute pairwise covariance using the same procedure described in the main text, with the modification that batch A corresponded to modules $M_1, M_2, M_4, M_6, M_9, M_{10}$ and batch B to modules $M_3, M_5, M_6, M_7, M_8, M_{10}$. This simulation corresponds to a design matrix with two covariates: intercept for the average co-expression and batch (0 for Batch A, 1 for Batch B). Our goal was to demonstrate that COBRA can discriminate between $\Sigma_{\text{batch A}}$ and $\Sigma_{\text{batch B}}$ in the aggregated gene expression data. We computed ROC curves between the batch-corrected co-expressions and $\Sigma_{\text{batch A}}$ using the same benchmarks as described in the main text (Figure S4), showing that, in contrast to naive methods and standard batch correction, COBRA is able to remove higher-order batch effects. We found an AUROC of 0.96 between $\Sigma_{\text{batch B}}$ and the COBRA component corresponding to batch B, providing further evidence that COBRA can identify and remove batch effects in gene co-expression.

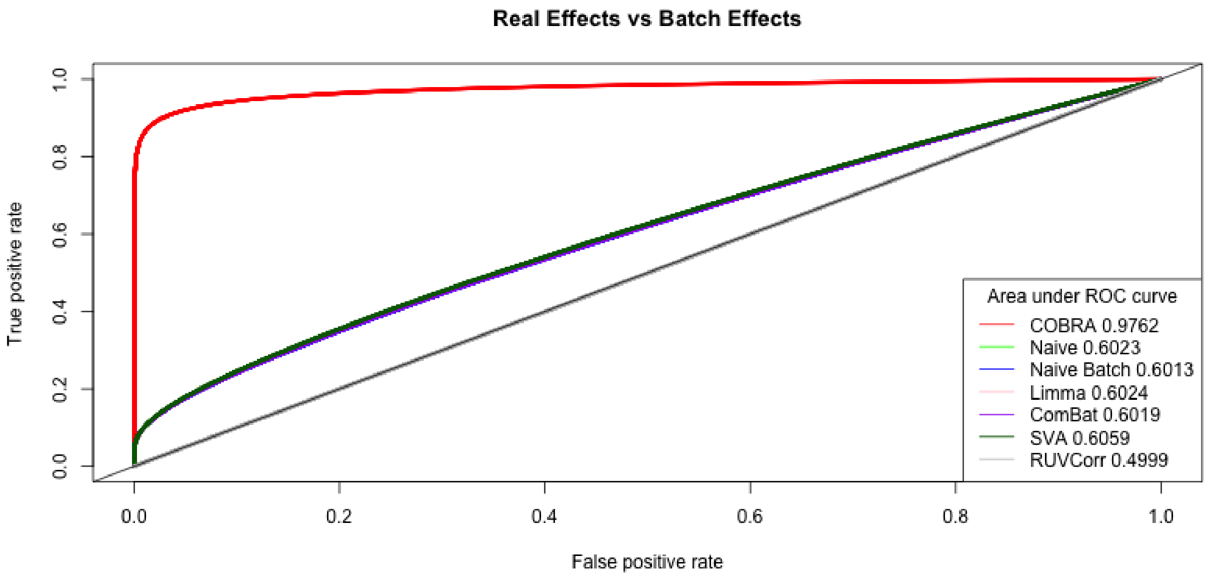

Figure S4: **Simulations demonstrate batch correction on gene co-expression.** ROC curves for each method provide a measure of its ability to identify $\Sigma_{\text{batch A}}$ using simulated *in silico* data containing batch effects.

Table S1: Percentage of co-expression outliers in COBRA components in thyroid cancer and ENCODE co-expression networks.

**Network**/ Component	Ratio of entries not in $[-1, 1]$	Minimum entry	Maximum entry
**Thyroid cancer**			
$\bar{C}$	0.02%	-1.09	1.38
Cancer	0%	-0.85	0.85
Sex	0%	-0.14	0.12
Age	0%	-0.004	0.01
Race 1	$4 \cdot 10^{-4}$%	-1.12	1.10
Race 2	0%	-0.39	0.40
Race 3	0%	-0.47	0.54
Race 4	0%	-0.58	-0.70
Race 5	0%	-0.43	0.46
Stage 1	$1.88 \cdot 10^{-5}$%	-1.22	1.36
Stage 2	0%	-0.88	0.56
Stage 3	0%	-0.61	0.52
Stage 4	0%	-0.91	0.76
Stage 5	5.9%	-4.03	5.35
Stage 6	$1.80 \cdot 10^{-6}$%	-1.06	0.88
Stage 7	0%	-0.38	0.78
Batch 1	0%	-0.40	0.55
Batch 2	0%	-0.42	0.59
Batch 3	0.15%	-1.07	0.74
Batch 4	0%	-0.48	0.76
Batch 5	0%	-0.35	0.82
Batch 6	0%	-0.71	0.77
Batch 7	0%	-0.53	0.53
Batch 8	0%	-0.22	0.43
Batch 9	0%	-0.38	0.67
Batch 10	0%	-0.41	0.29
Batch 11	0%	-0.29	0.47
Batch 12	0.45%	-1.13	1.02
Batch 13	0%	-0.24	0.19
Batch 14	0%	-0.37	0.36
Bath 15	0%	-0.37	0.47
Batch 16	0.32%	-1.12	0.73
Batch 17	0%	-0.57	0.61
**ENCODE (0.5 vs 0.25 proportion)**			
$\bar{C}$	0.0008%	-0.92	1.16
Group	0%	-0.19	0.16
Batch	0%	-0.32	0.25

Table S2: Runtimes for computing batch corrected differential co-expression in the experiments presented in the *Improved co-expression estimates* in silico section in the main text. Measured on an Apple M2 Pro laptop with 16GB RAM.

**Method**	Runtime (in seconds)
COBRA	17.3s
Naive	2.57s
Naive batch	2.75s
Limma	2.62s
RUV Cor	3.46s
ComBat	5.55s
SVA	96.8s

Table S3: Runtimes of computing a COBRA batch corrected co-expression for THCA gene expression data from TCGA on a random set of genes with the given size. We also included the runtime to compute gene co-expression without batch correction as a baseline. Measured on an r5.2xlarge with 64GB RAM.

**Number of genes**	Runtime	Baseline
10^3 genes	5.4s	4.2s
10^4 genes	122.4s	5.9s
$2 \cdot 10^4$ genes	430.8s	24.89s
$2.2 \cdot 10^4$ genes	656.4s	39.76s

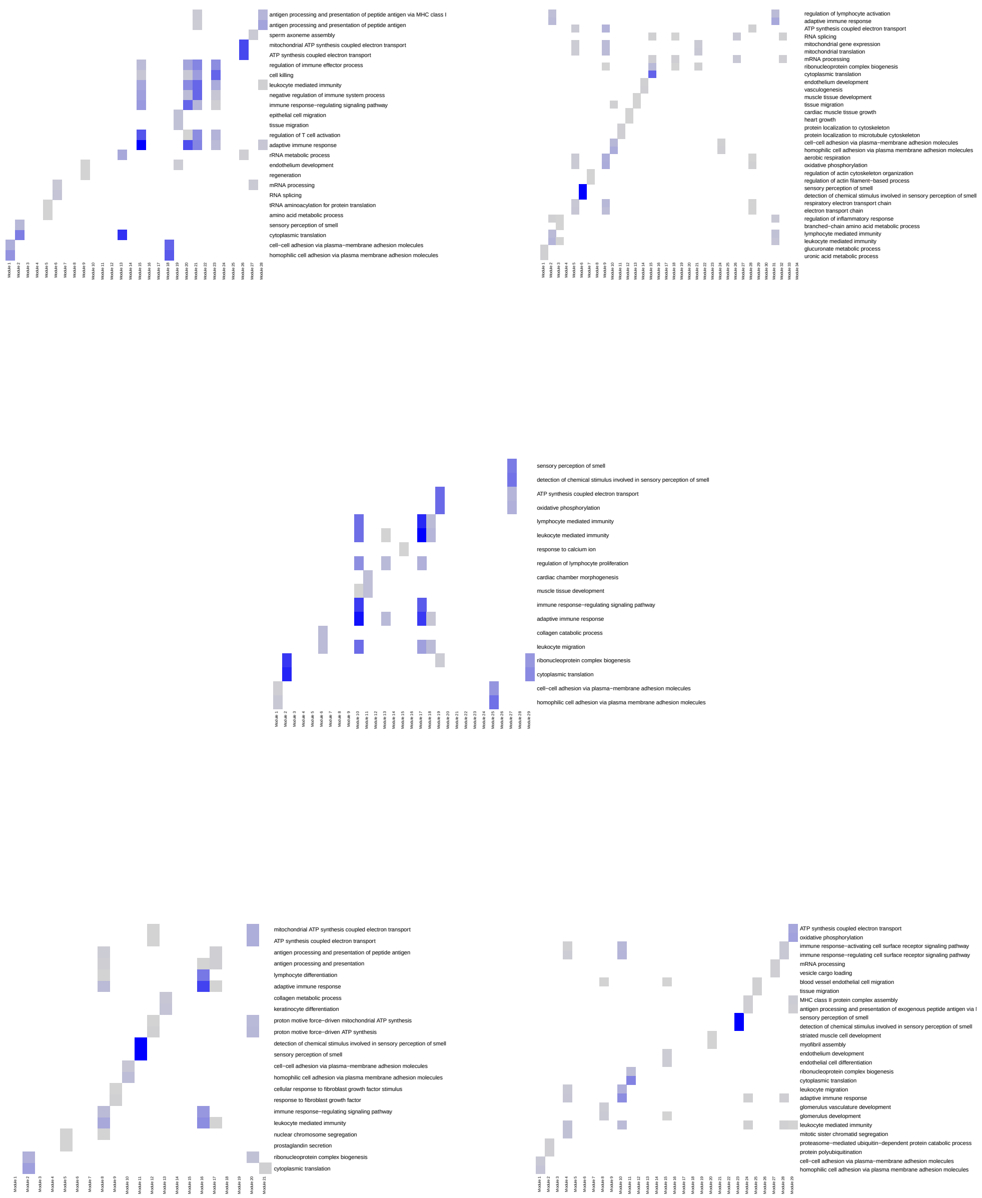

Figure S5: **Recurrence analysis of the thyroid cancer pipeline on GO.** Gene set enrichment analysis using Gene Ontology annotations for thyroid cancer co-expression networks using five bootstrapped subsets of the full dataset.

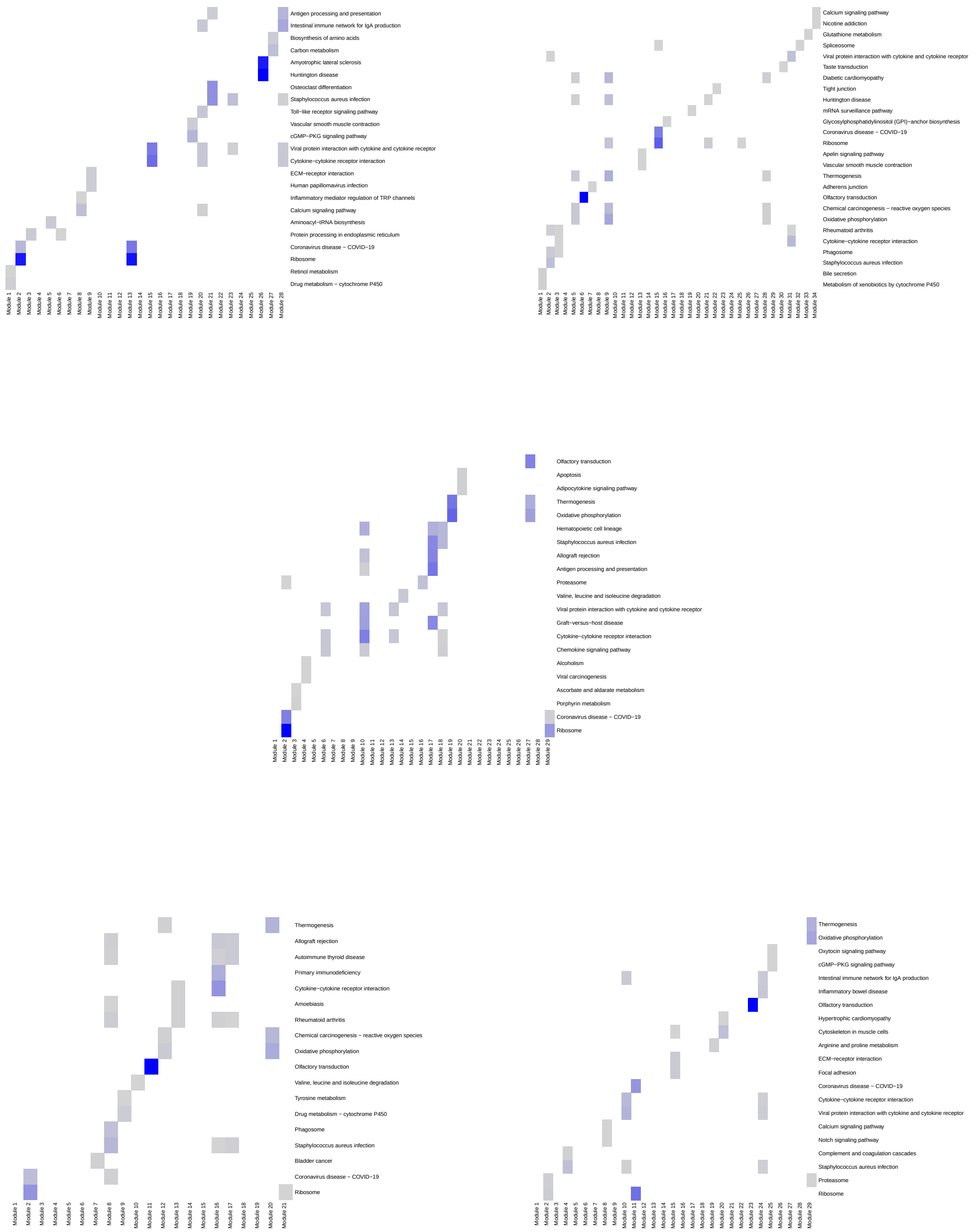

Figure S6: **Recurrence analysis of the thyroid cancer pipeline on KEGG.** Gene set enrichment analysis using KEGG annotations for thyroid cancer co-expression networks using five bootstrapped subsets of the full dataset.

References

[Ben Guebila et al., 2022] Ben Guebila, M., Lopes-Ramos, C. M., Weighill, D., Sonawane, A. R., Burkholz, R., Shamsaei, B., Platig, J., Glass, K., Kuijjer, M. L., and Quackenbush, J. (2022). Grand: a database of gene regulatory network models across human conditions. *Nucleic Acids Research*, 50(D1):D610–D621.

[GTEx-Consortium, 2015] GTEx-Consortium (2015). The genotype-tissue expression (gtex) pilot analysis: multitissue gene regulation in humans. *Science*, 348(6235):648–660.
